# Supplementary material for: CDC20 promotes bone formation via APC/C dependent ubiquitination and degradation of p65
Source: EMBO Rep. 2021 Aug 12;22(9):e52576. doi: 10.15252/embr.202152576 (PMC8419691; doi:10.15252/embr.202152576)
Supplement: Supplementary file 2 — Appendix [file EMBR-22-e52576-s005.pdf]

Appendix

Appendix Figure S1---2

Appendix Figure S2---4

Appendix Figure S3---6

Appendix Figure S4---8

Appendix Figure S5---10

Appendix Figure S6---12

Appendix Figure S7---13

Appendix Figure S8---15

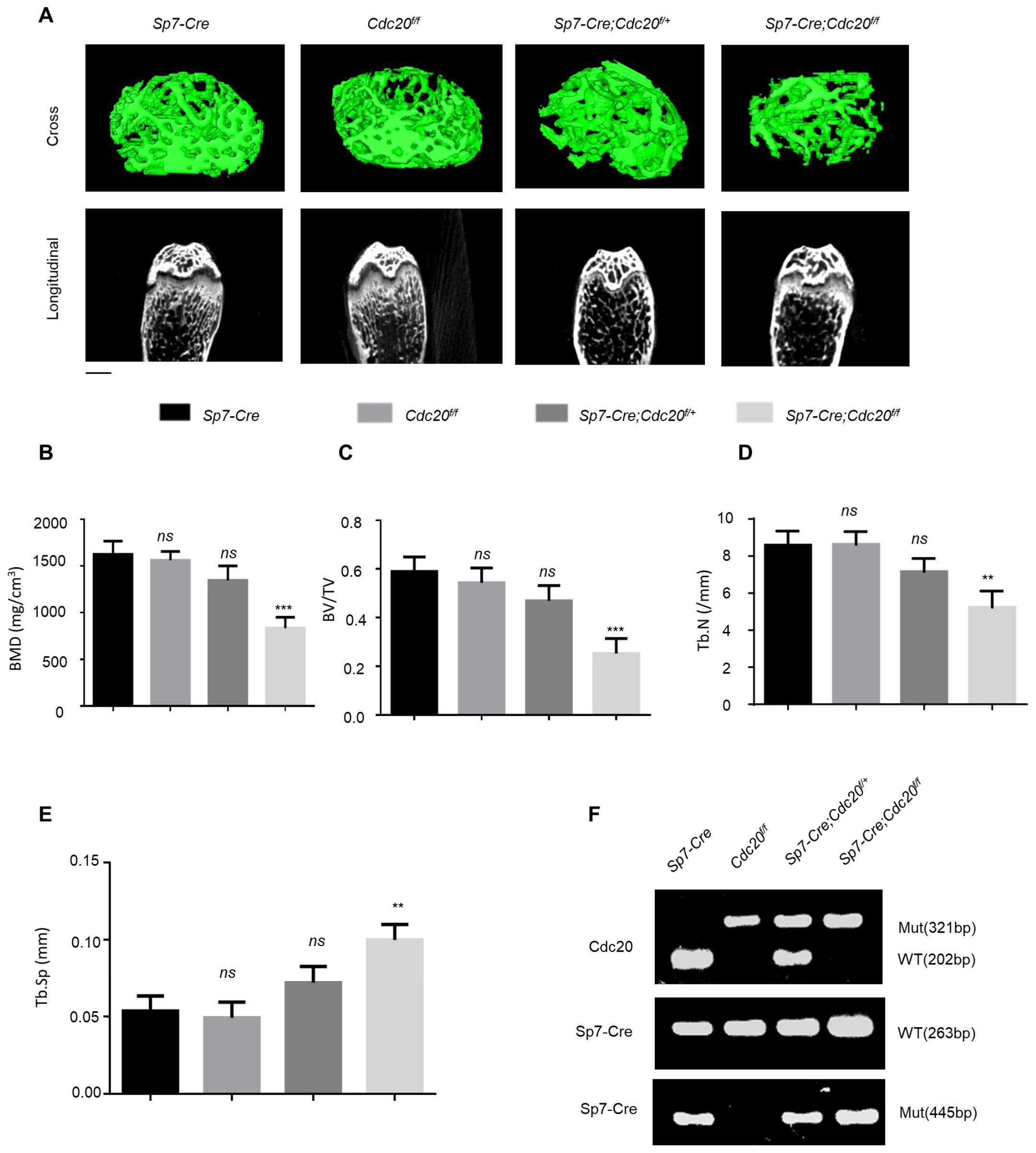

Appendix Figure S1

(A) Representative micro-CT images of trabecular bone from the femoral metaphysis of 6-week-old *Sp7-Cre, Cdc20*$^{f/f}$, *Sp7-Cre;Cdc20*$^{f/+}$ and *Sp7-Cre;Cdc20*$^{f/f}$ mice. Scale bar, 500 μm.

(B-E) Histomorphometric analyses of 6-week-old femurs. (n=3)

(F) Representative image of PCR genotypes of *Sp7-Cre, Cdc20*$^{f/f}$, *Sp7-Cre;Cdc20*$^{f/+}$ and *Sp7-Cre;Cdc20*$^{f/f}$ mice.

Data information: Data are displayed as mean ± SD and show one representative of n ≥ 3 independent experiments with three biological replicates. Statistical significance was calculated by one-way ANOVA followed by a Tukey's post hoc test and defined as *ns*, *not significant*, $^{**}p<0.01$, $^{***}p<0.001$.

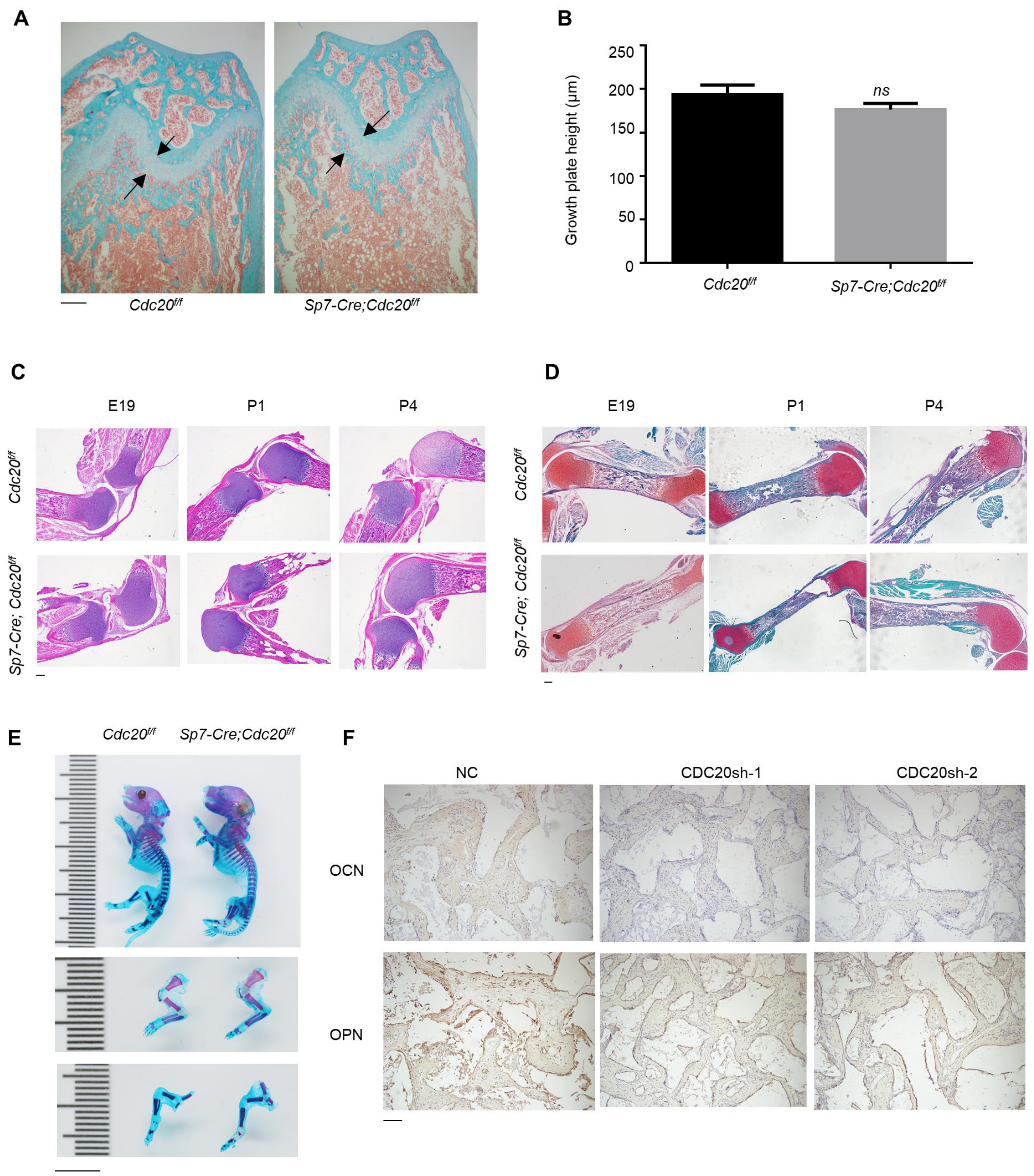

Appendix Figure S2

(A) Representative Goldner's trichrome staining of chondrocytes from the femoral metaphysis of 6-week-old *Cdc20*$^{f/f}$ and *Sp7-Cre;Cdc20*$^{f/f}$ mice. Scale bar, 200 µm.

(B) Statistical analyses of growth plate height of 6-week-old mice femurs. (n=6)

(C, D) Representative H&E staining and Safranin-O-Fast Green staining of femurs and tibea of embryonic day 19 (E19), postnatal day 1 (P1) and day 4 (P4) of *Cdc20*$^{f/f}$ and *Sp7-Cre;Cdc20*$^{f/f}$ mice. Scale bar, 200 μm.

(E) Whole-mount skeleton staining of *Cdc20*$^{f/f}$ and *Sp7-Cre;Cdc20*$^{f/f}$ P2 mice. The upper one shows the whole skeleton. The middle one shows the upper extremities, and the lower one shows the hind limbs of *Cdc20*$^{f/f}$ and *Sp7-Cre;Cdc20*$^{f/f}$ mice. Scale bar, 1cm.

(F) The expression of OCN and OPN in sections of NC and CDC20sh scaffolds using IHC assay. Scale bar, 50 μm.

Data information: Data are displayed as mean ± SD and show one representative of n ≥ 3 independent experiments with three biological replicates. Statistical significance was calculated by a two-tailed unpaired Student's *t*-test and defined as *ns*, *not significant*.

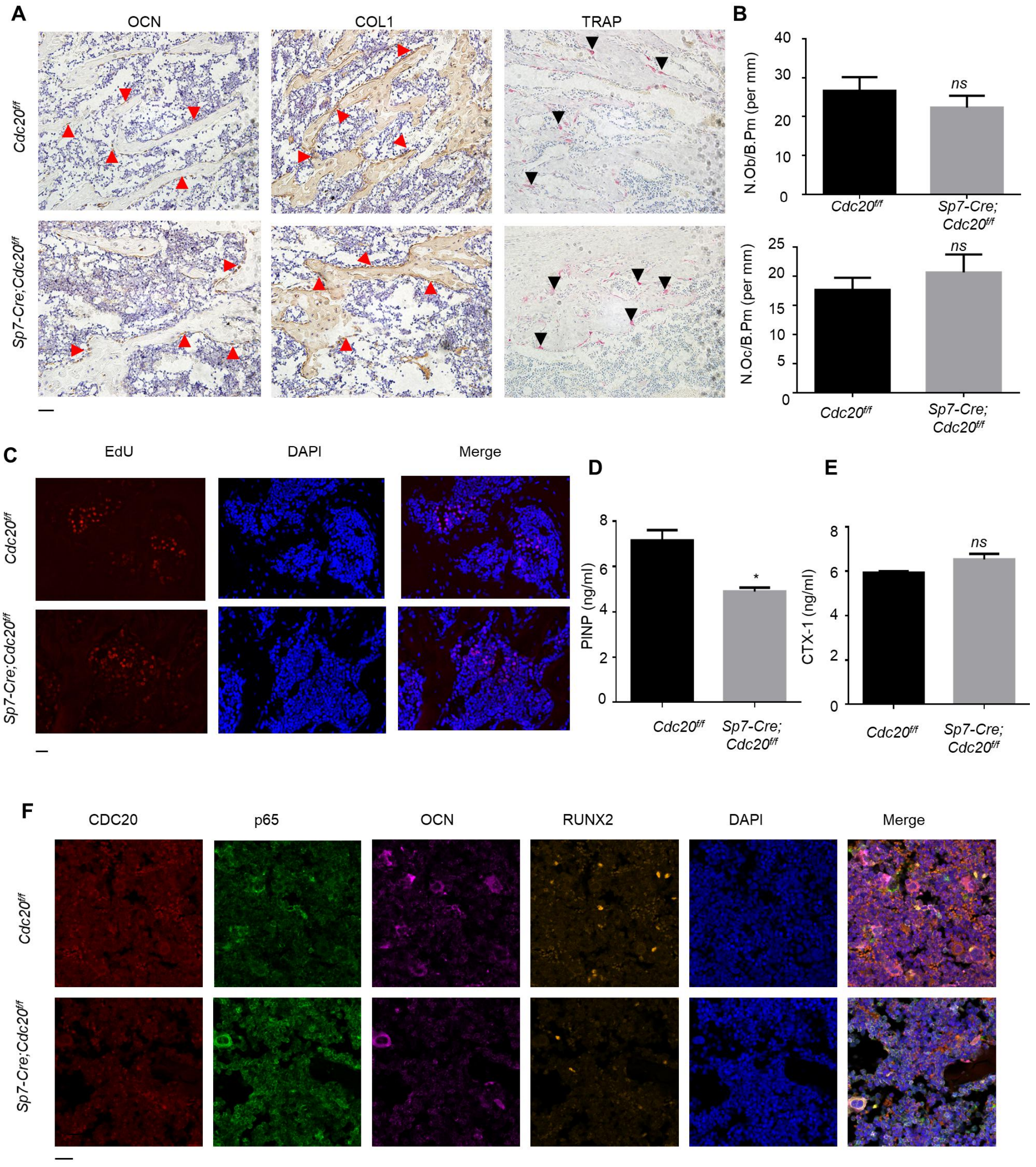

Appendix Figure S3

(A) Immunohistochemistry of OCN and COL1, and TRAP staining of 6-week-old *Cdc20$^{f/f}$*, *Sp7-Cre;Cdc20$^{f/f}$* mice femurs. The arrows represent the positive expression cells. Scale bar, 50 μm.

(B) Measurements and statistical analyses of numbers of osteoblasts and osteoclasts in 6-week-old *Cdc20$^{f/f}$* and *Sp7-Cre;Cdc20$^{f/f}$* mice femurs. (n=6)

(C) Immunofluorescence of EdU of 6-week-old *Cdc20*$^{f/f}$*, Sp7-Cre;Cdc20*$^{f/f}$ mice femurs. Scale bar, 50 μm.

(D, E) Measurements and statistical analyses of PINP and CTX-1 in the serum of 6-week-old *Cdc20*$^{f/f}$ and *Sp7-Cre;Cdc20*$^{f/f}$ mice. (n=6)

(F) Immunofluorescence of relative expression of CDC20, p65, OCN, RUNX2 on 6-week-old *Cdc20*$^{f/f}$ and *Sp7-Cre;Cdc20*$^{f/f}$ mice femurs. Scale bar, 20 μm.

Data information: Data are displayed as mean ± SD and show one representative of n ≥ 3 independent experiments with three biological replicates. Statistical significance was calculated by a two-tailed unpaired Student's *t*-test and defined as *ns*, *not significant*, *p<0.05. N.Ob/B.Pm (osteoblast number/bone perimeter), N.Oc/B.Pm (osteoclast number/bone perimeter).

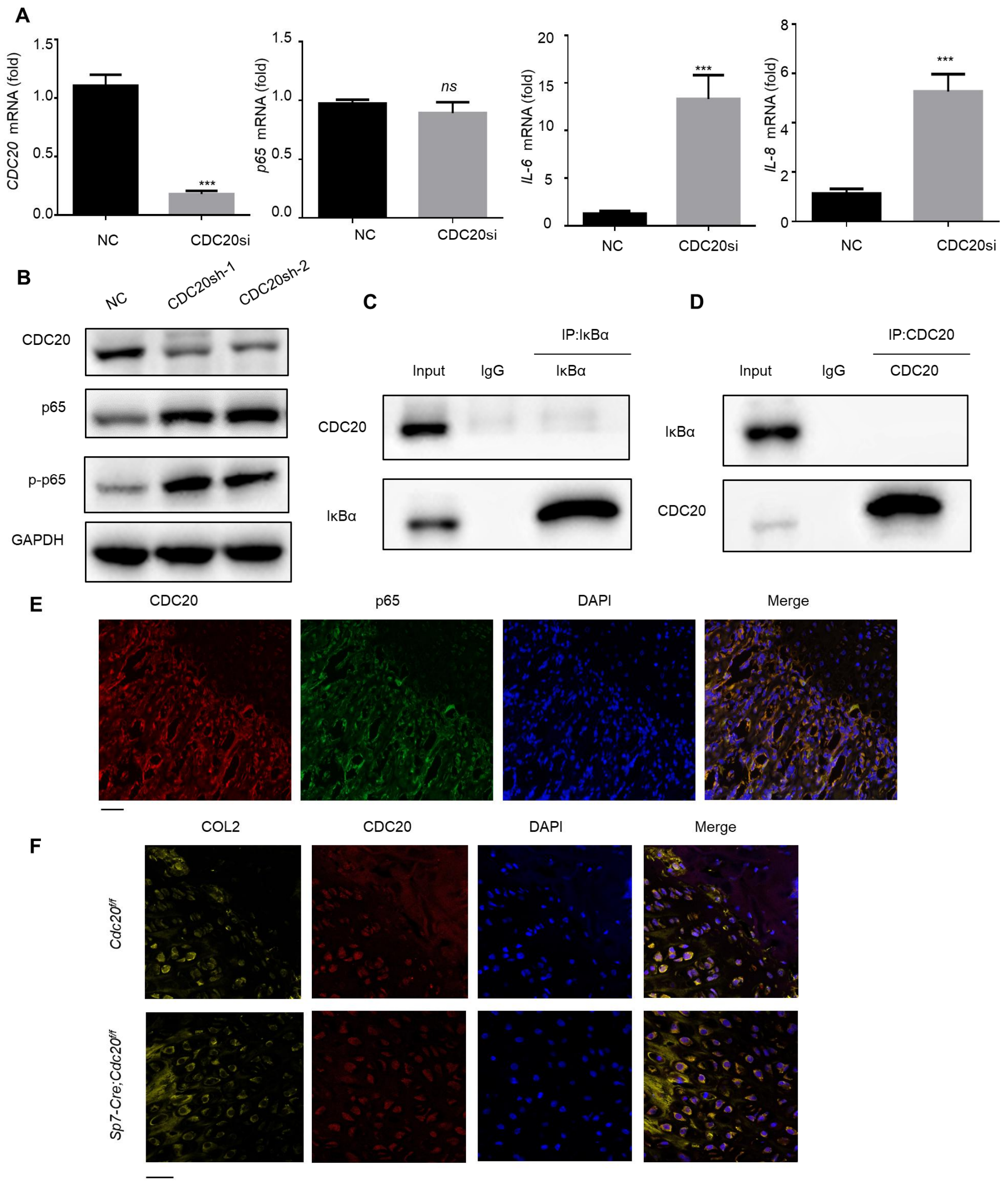

Appendix Figure S4

(A) The expression of *CDC20*, *p65* and NF-κB pathway downstream genes *IL-6, IL-8* of NC and CDC20si hBMSCs determined by qRT-PCR. (n=5)

(B) Western blot analyses of p65, p-p65 in NC and CDC20sh hBMSCs.

(C, D) No interaction was found between CDC20 and IκBα in hBMSCs.

(E) The co-localization of CDC20 and p65 on 6-week-old bone sections. Scale bar, 50 μm.

(F) Immunofluorescence of relative expression of COL2 and CDC20 of 6-week-old *Cdc20*$^{f/f}$ and *Sp7-Cre;Cdc20*$^{f/f}$ mice femurs. Scale bar, 20 μm.

Data information: Data are displayed as mean ± SD and show one representative of n ≥ 3 independent experiments with three biological replicates. Statistical significance was calculated by a two-tailed unpaired Student's *t*-test and defined as *ns*, *not significant*; ***p<0.001.

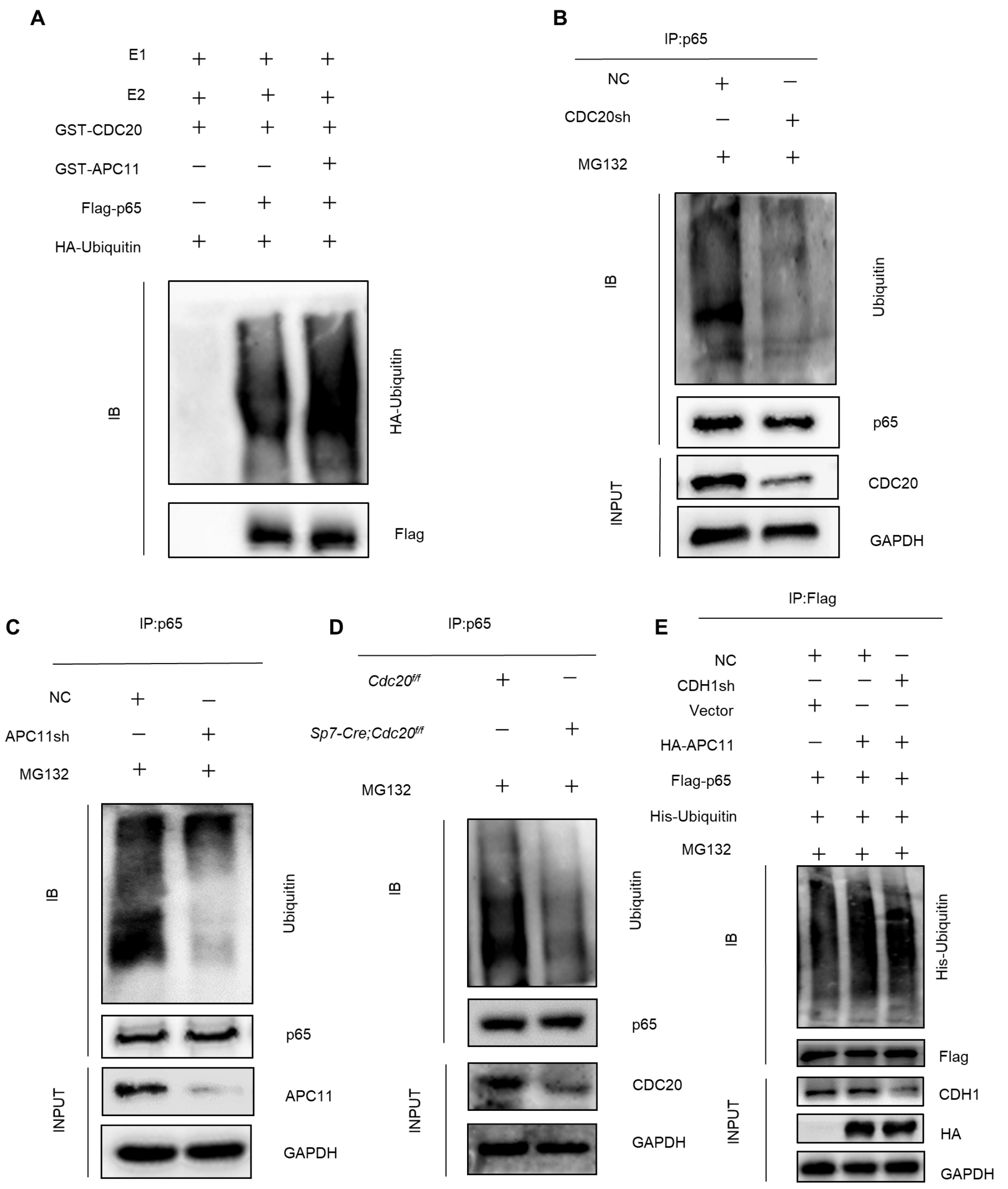

Appendix Figure S5

(A) Immunoblot of Flag-p65-linked in vitro ubiquitination promoted by GST-CDC20 or GST-CDC20 with GST-APC11. Bacterially expressed and purified GST-CDC20 or GST-CDC20 with GST-APC11 were incubated with purified proteins, including HA-ubiquitin, E1, E2, and Flag-p65 from cell lysis at 32 °C for 1 h.

(B) Immunoblot of p65-linked ubiquitination after the knockdown of CDC20. NC and CDC20sh HEK293T cells were treated with 10 μM MG132 for 6 h, whole cell protein extracts were immunoprecipitated with anti-p65 and immunoprecipitation was immunoblotted for Ubiquitin.

(C) Immunoblot of p65-linked ubiquitination after the knockdown of APC11. NC and APC11sh HEK293T cells were treated with 10 μM MG132 for 6 h, whole cell protein extracts were immunoprecipitated with anti-p65 and immunoprecipitation was immunoblotted for Ubiquitin.

(D) Immunoblot of endogenous p65-linked ubiquitination. BMSCs of 6-week-old *Cdc20*$^{f/f}$ and *Sp7-Cre;Cdc20*$^{f/f}$ mice were treated with 10 μM MG132 for 6 h before collection.

(E) Immunoblot of Flag-p65-linked ubiquitination promoted by HA-APC11 in NC and CDH1sh cells. NC or CDH1sh HEK293T cells were co-transfected with Flag-p65, His-Ubiquitin, with or without HA-APC11 and Vector plasmids. Transfected cells were then treated with 10 μM MG132 for 6 h before collection.

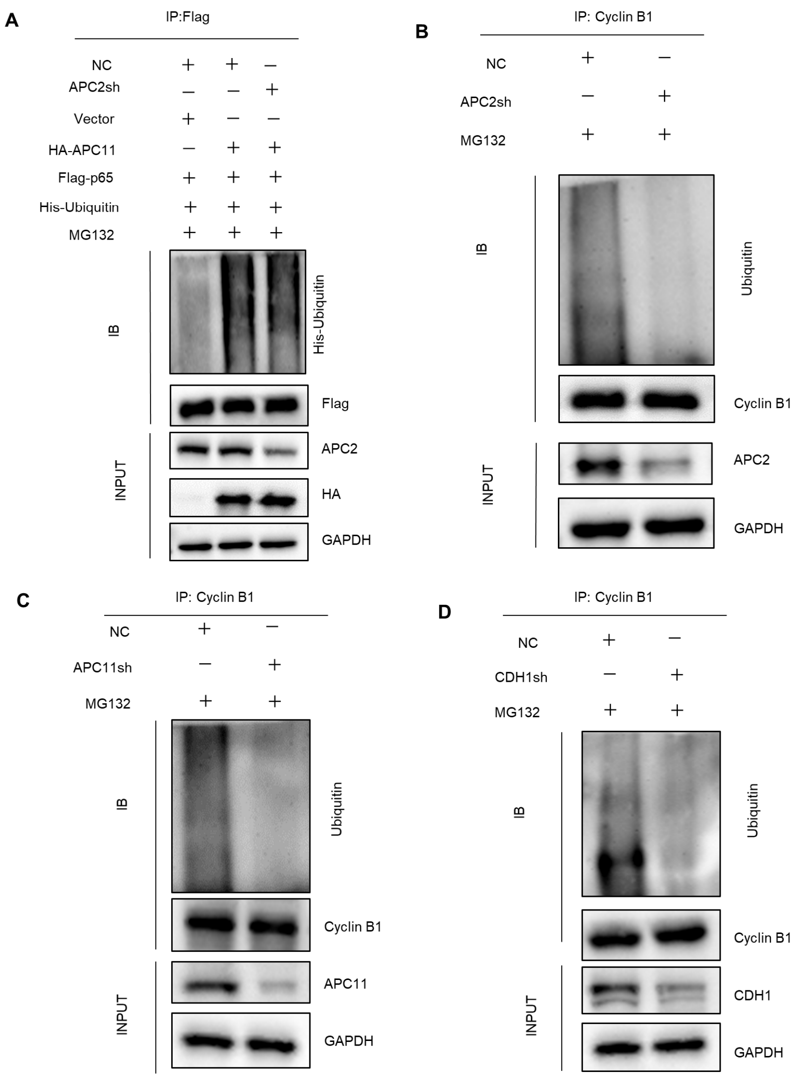

Appendix Figure S6

(A) NC or APC2sh HEK293T cells were co-transfected with Flag-p65, His-Ubiquitin, with or without HA-APC11 and Vector plasmids. Transfected cells were then treated with 10 μM MG132 for 6 h before collection.

(B-D) NC, APC2sh, APC11sh, CDH1sh HEK293T cells were treated with 10 μM MG132 for 6 h, whole cell protein extracts were immunoprecipitated with anti-Cyclin B1 and immunoprecipitations were immunoblotted for Ubiquitin.

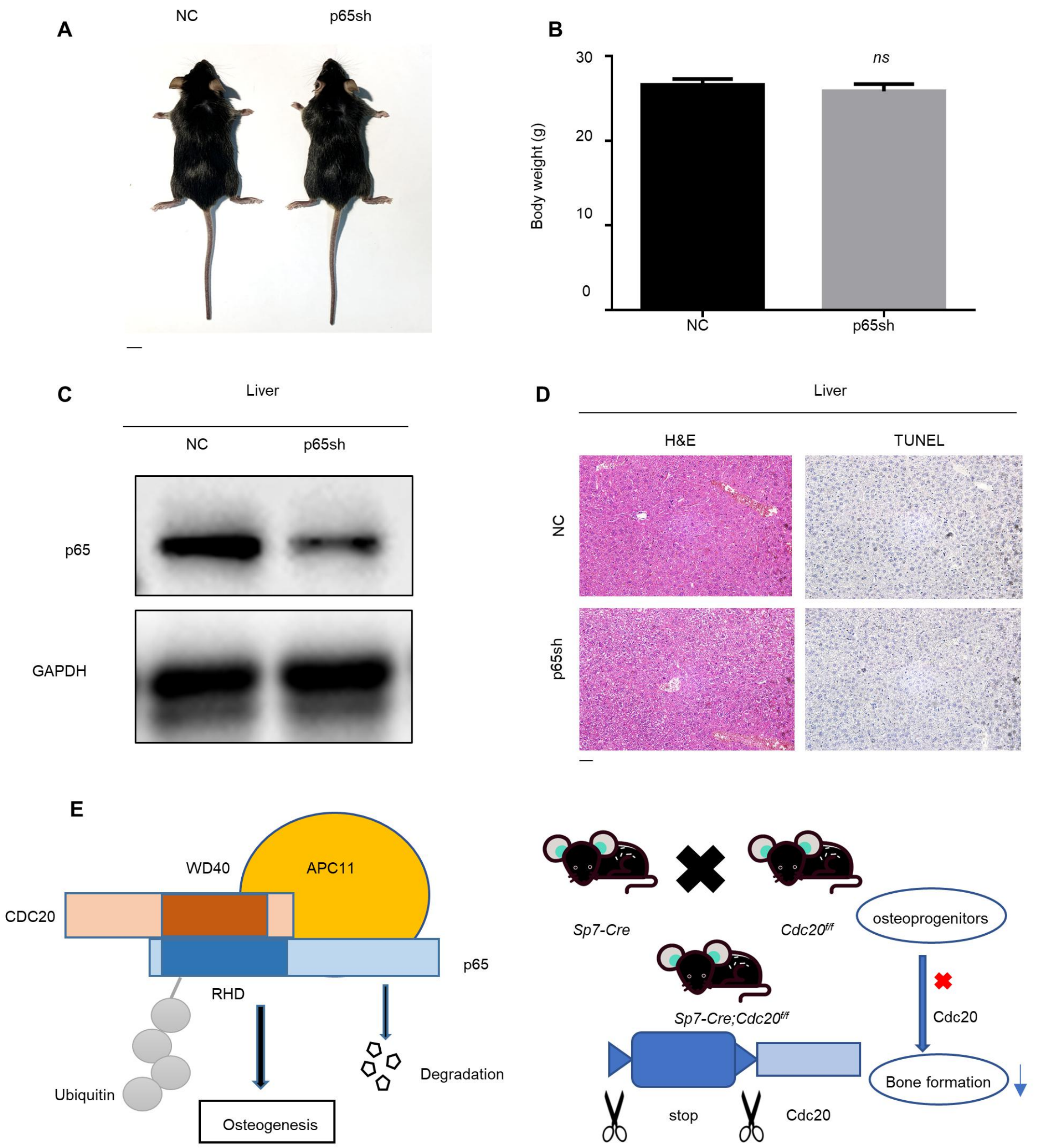

Appendix Figure S7

(A, B) The appearance and body weight of NC and p65sh lentivirus injected mice. Scale bar, 1cm.

(C) Western blot of p65 knockdown in the liver of NC and p65sh mice.

(D) The H&E staining and TUNEL staining in the liver of NC and p65sh mice. Scale bar: 50μm.

(E) A model depicting how CDC20 regulates bone formation through p65. The left model shows

the relationship of APC11, CDC20, p65 and ubiquitin. The right model shows the method to generate the mice and loss of Cdc20 in osteoprogenitors in mice results in decreased bone formation. Data information: Data are displayed as mean ± SD and show one representative of n ≥ 3 independent experiments with three biological replicates. Statistical significance was calculated by a two-tailed unpaired Student's *t*-test and defined as *ns*, *not significant*

.

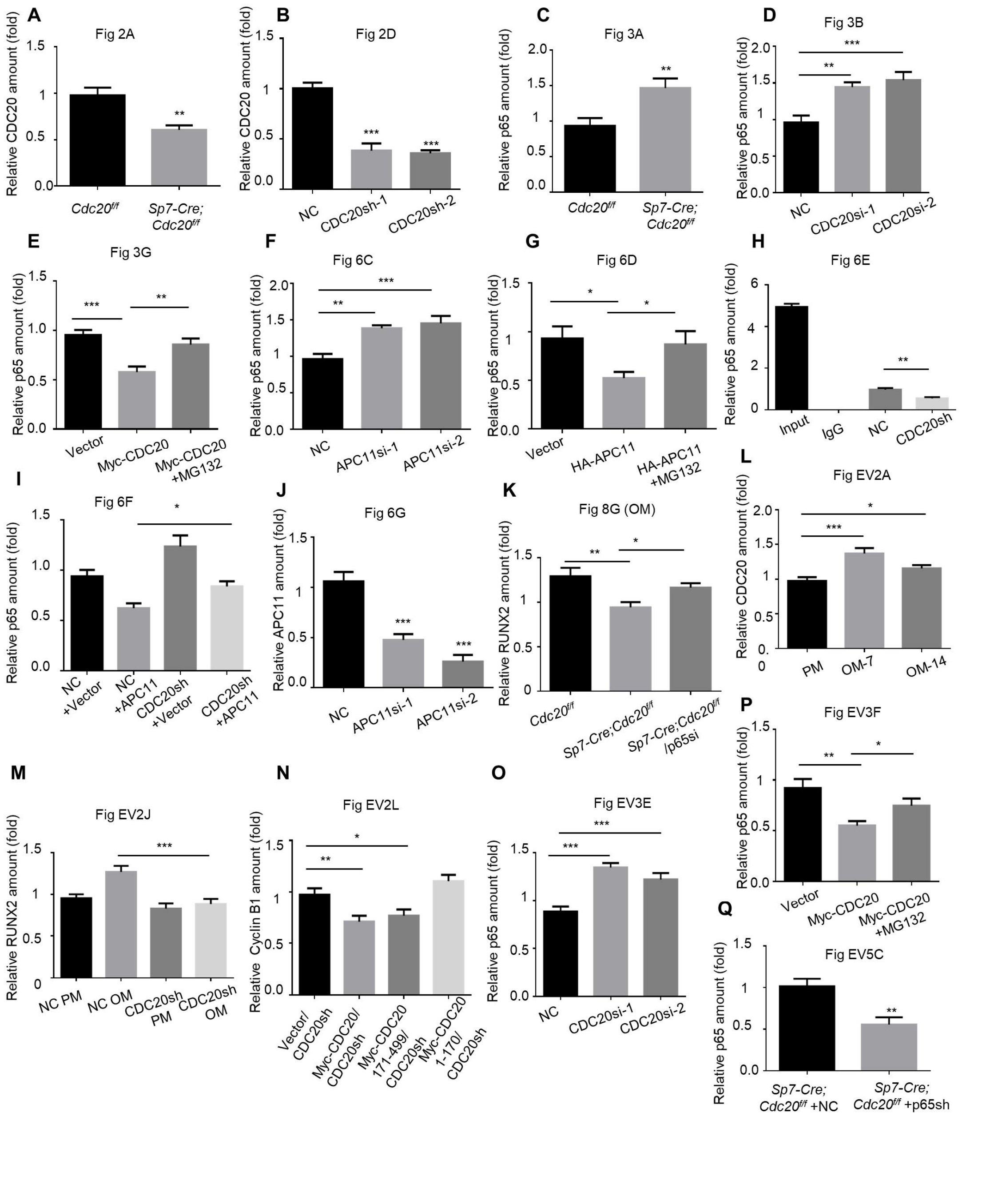

Appendix Figure S8

(A-Q) Quantifications and statistical analyses of the significant differences in western blot experiments. Data information: Data are displayed as mean ± SD and show one representative of n ≥ 3 independent experiments with three biological replicates. Statistical significance was calculated by a two-tailed unpaired Student's *t*-test or one-way ANOVA followed by a Tukey's post hoc test and defined as **p*<0.05; ***p*<0.01; ****p*<0.001.
