# Supplementary material for: CDC20 promotes bone formation via APC/C dependent ubiquitination and degradation of p65
Source: EMBO Rep. 2021 Aug 12;22(9):e52576. doi: 10.15252/embr.202152576 (PMC8419691; doi:10.15252/embr.202152576)
Supplement: Supplementary file 3 — Expanded View Figures PDF [file EMBR-22-e52576-s003.pdf]

Expanded View Figures

Figure EV1. Conditional knockout of Cdc20 impairs bone formation and blunts bone regeneration, related to Fig 1.

A The design strategy of conditional deletion of Cdc20 gene.
B Representative image of PCR genotypes of indicated mice. *Sp7-Cre;Cdc20*$^{f/f}$ mice were in experimental groups, *Cdc20*$^{f/f}$ mice were in control groups.
C Representative micro-CT images and H&E staining of trabecular bone from the femoral metaphysis of 12-week-old male *Sp7-Cre;Cdc20*$^{f/f}$ and littermate control mice. Scale bar, 500 μm.
D Histomorphometric analyses of 12-week-old male femurs (n = 6).
E Representative micro-CT images and H&E staining of trabecular bone from the femoral metaphysis of 12-week-old female *Sp7-Cre;Cdc20*$^{f/f}$ and littermate control mice. Scale bar, 500 μm.
F Histomorphometric analyses of 12-week-old female femurs (n = 6).

Data information: Data are displayed as mean ± SD and show one representative of $n \geq 3$ independent experiments with three biological replicates. Statistical significance was calculated by a two-tailed unpaired Student's t-test and defined as $^*P < 0.05$, $^{**}P < 0.01$.

 © 2021 The Authors

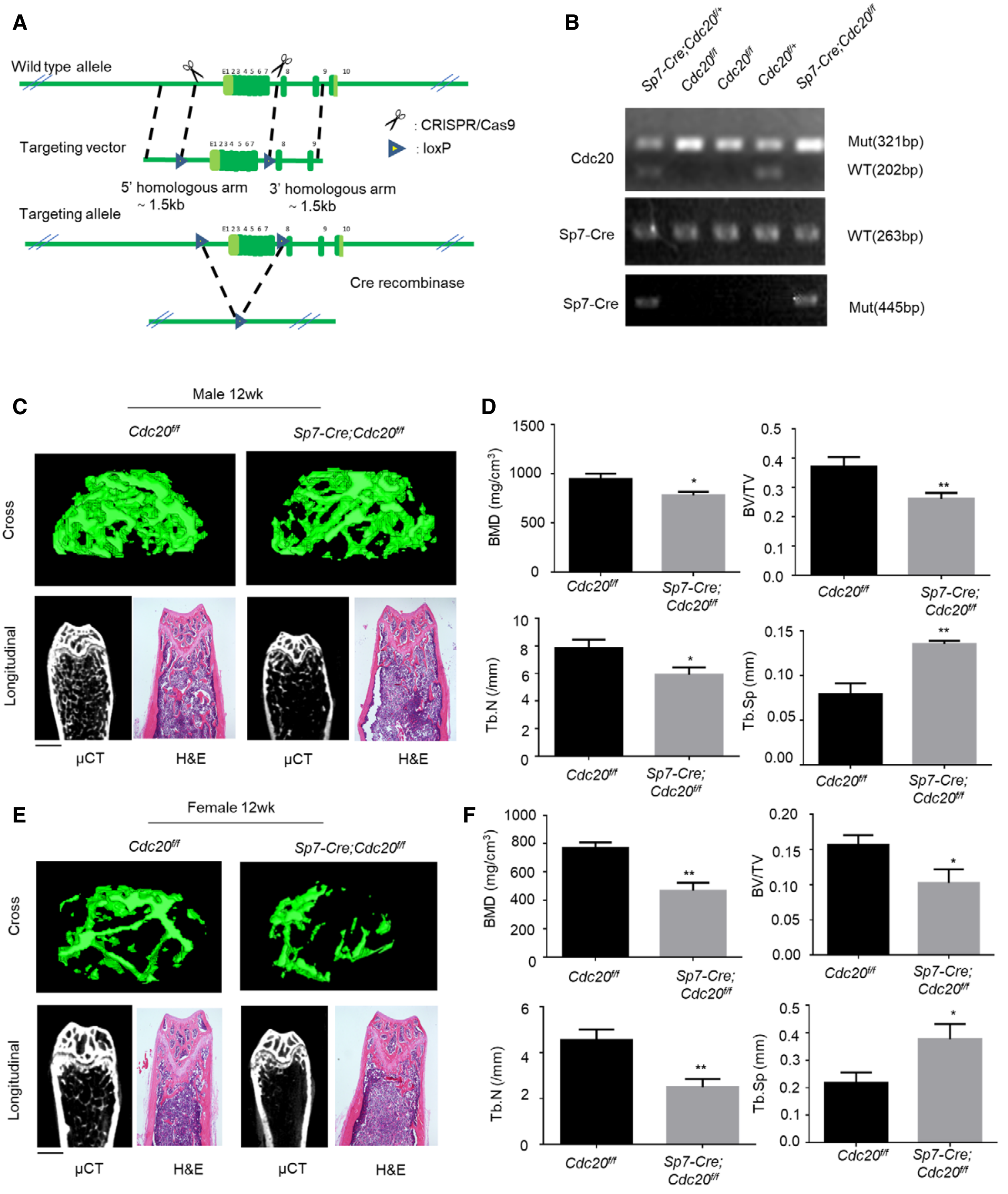
A
Wild type allele
Targeting vector
5' homologous arm
~ 1.5kb
3' homologous arm
~ 1.5kb
Targeting allele
: CRISPR/Cas9
: loxP
Cre recombinase
B
Sp7-Cre;Cdc20f/+
Cdc20f/f
Cdc20f/f
Cdc20f/+
Sp7-Cre;Cdc20f/f
Cdc20
Mut(321bp)
WT(202bp)
Sp7-Cre
WT(263bp)
Sp7-Cre
Mut(445bp)
C
Male 12wk
Cdc20f/f
Sp7-Cre;Cdc20f/f
Cross
Longitudinal
µCT
H&E
µCT
H&E
D
BMD (mg/cm3)
BV/TV
Tb.N (/mm)
Tb.Sp (mm)
Cdc20f/f
Sp7-Cre; Cdc20f/f
E
Female 12wk
Cdc20f/f
Sp7-Cre;Cdc20f/f
Cross
Longitudinal
µCT
H&E
µCT
H&E
F
BMD (mg/cm3)
BV/TV
Tb.N (/mm)
Tb.Sp (mm)
Cdc20f/f
Sp7-Cre; Cdc20f/f

Figure EV1.

Figure EV2. CDC20 modulates osteogenic differentiation of BMSCs, related to Fig 2.

A–D Western blot analyses (A) and qRT–PCR (B-D) of the expression of CDC20 and osteogenic marker RUNX2, OCN. Cells were cultured in osteogenic medium for 7 and 14 days (n = 6).

E, F The knockout efficiency of *Cdc20* (E) and the expression of osteogenic marker *Runx2* (F) in BMSCs of *Sp7-Cre;Cdc20$^{f/f}$* and *Cdc20$^{f/f}$* mice determined by qRT–PCR (n = 5).

G Representative images of light and fluorescence of lentivirus infected NC and CDC20sh hBMSCs. Scale bar: 500 μm.

H The knockdown efficiency of *CDC20* in NC and CDC20sh hBMSCs determined by qRT–PCR (n = 5).

I, J The expression of RUNX2 in NC and CDC20sh hBMSCs after 7 days osteogenic differentiation determined by qRT–PCR (I) and Western blot analyses (J) (n = 5).

K Western blot analyses of Myc-CDC20, Myc-CDC20 171–499 fragment (containing WD40 domain), Myc-CDC20 1–170 fragment (lacking WD40 domain) plasmids expression in HEK293T cells.

L Western blot analyses of the degradation of the substrate Cyclin B1 under the overexpression of truncated fragments of CDC20.

Data information: Data are displayed as mean ± SD and show one representative of $n \geq 3$ independent experiments with three biological replicates. Statistical significance was calculated by a two-tailed unpaired Student's t-test or one-way ANOVA followed by a Tukey's post hoc test and defined as ***$P < 0.001$.

 © 2021 The Authors

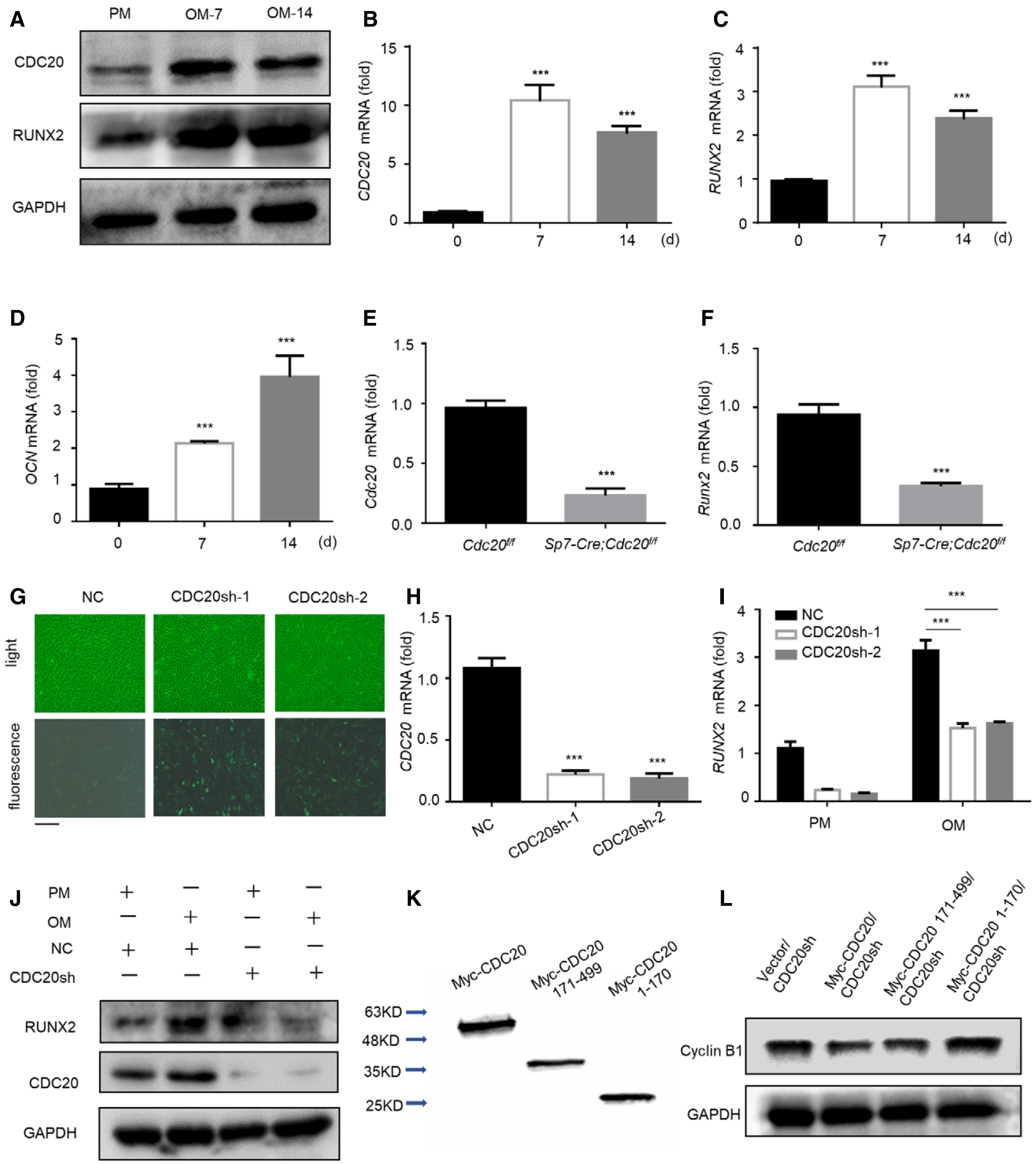
A
PM
OM-7
OM-14
CDC20
RUNX2
GAPDH
B
CDC20 mRNA (fold)
0
7
14
(d)
C
RUNX2 mRNA (fold)
D
OCN mRNA (fold)
E
Cdc20 mRNA (fold)
Cdc20f/f
Sp7-Cre;Cdc20f/f
F
Runx2 mRNA (fold)
G
NC
CDC20sh-1
CDC20sh-2
light
fluorescence
H
CDC20 mRNA (fold)
I
RUNX2 mRNA (fold)
PM
OM
J
PM
OM
NC
CDC20sh
K
Myc-CDC20
Myc-CDC20 171-499
Myc-CDC20 1-170
63KD
48KD
35KD
25KD
L
Vector/ CDC20sh
Myc-CDC20/ CDC20sh
Myc-CDC20 171-499/ CDC20sh
Myc-CDC20 1-170/ CDC20sh
Cyclin B1
GAPDH

Figure EV2.

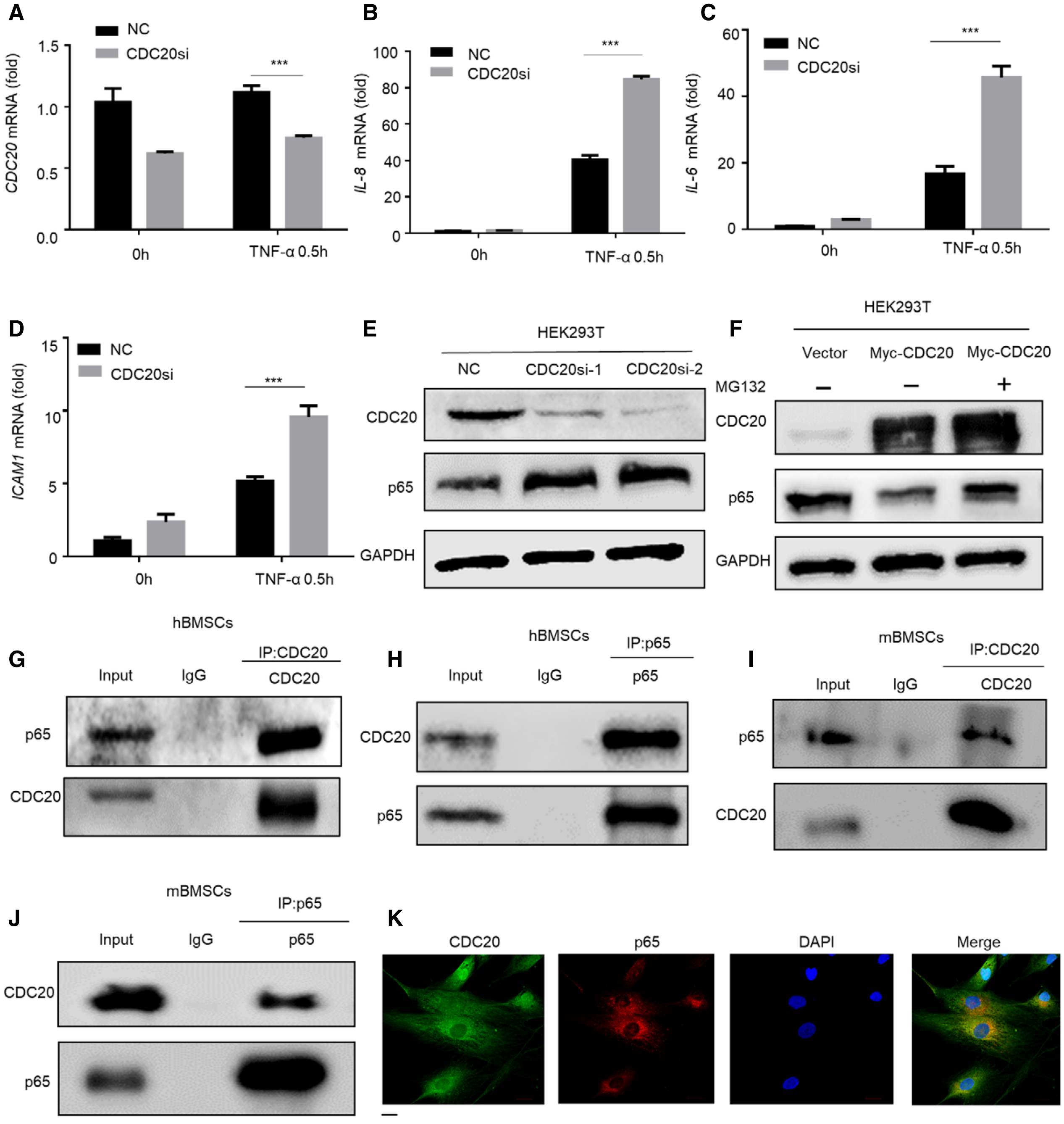

Figure EV3. CDC20 induces proteasome-dependent degradation of p65 and CDC20 interacts with p65, related to Figs 3 and 4.

A–D The expression of *CDC20* (A) and NF-κB pathway downstream genes *IL-8*, *IL-6*, and *ICAM1* (B-D) of NC and CDC20si HEK293T cells after TNF-α stimulation determined by qRT–PCR ($n = 6$).

E Western blot analyses of the degradation of endogenous p65 protein in NC and CDC20si HEK293T cells.

F Western blot analyses of the degradation of p65 protein after the overexpression of Myc-CDC20. HEK293T cells were transfected with Vector and Myc-CDC20 plasmids for 36 h, cells were treated with or without 10 μM MG132 (the proteasome inhibitor) for 6 h before collected.

G, H Co-immunoprecipitation of endogenous CDC20 with endogenous p65 in hBMSCs.

I, J Co-immunoprecipitation of endogenous CDC20 with endogenous p65 in mBMSCs.

K The co-localization of CDC20 and p65 in hBMSCs. Scale bar: 20 μm.

Data information: Data are displayed as mean ± SD and show one representative of $n \geq 3$ independent experiments with three biological replicates. Statistical significance was calculated by one-way ANOVA followed by a Tukey's post hoc test and defined as $^{***}P < 0.001$.

 © 2021 The Authors

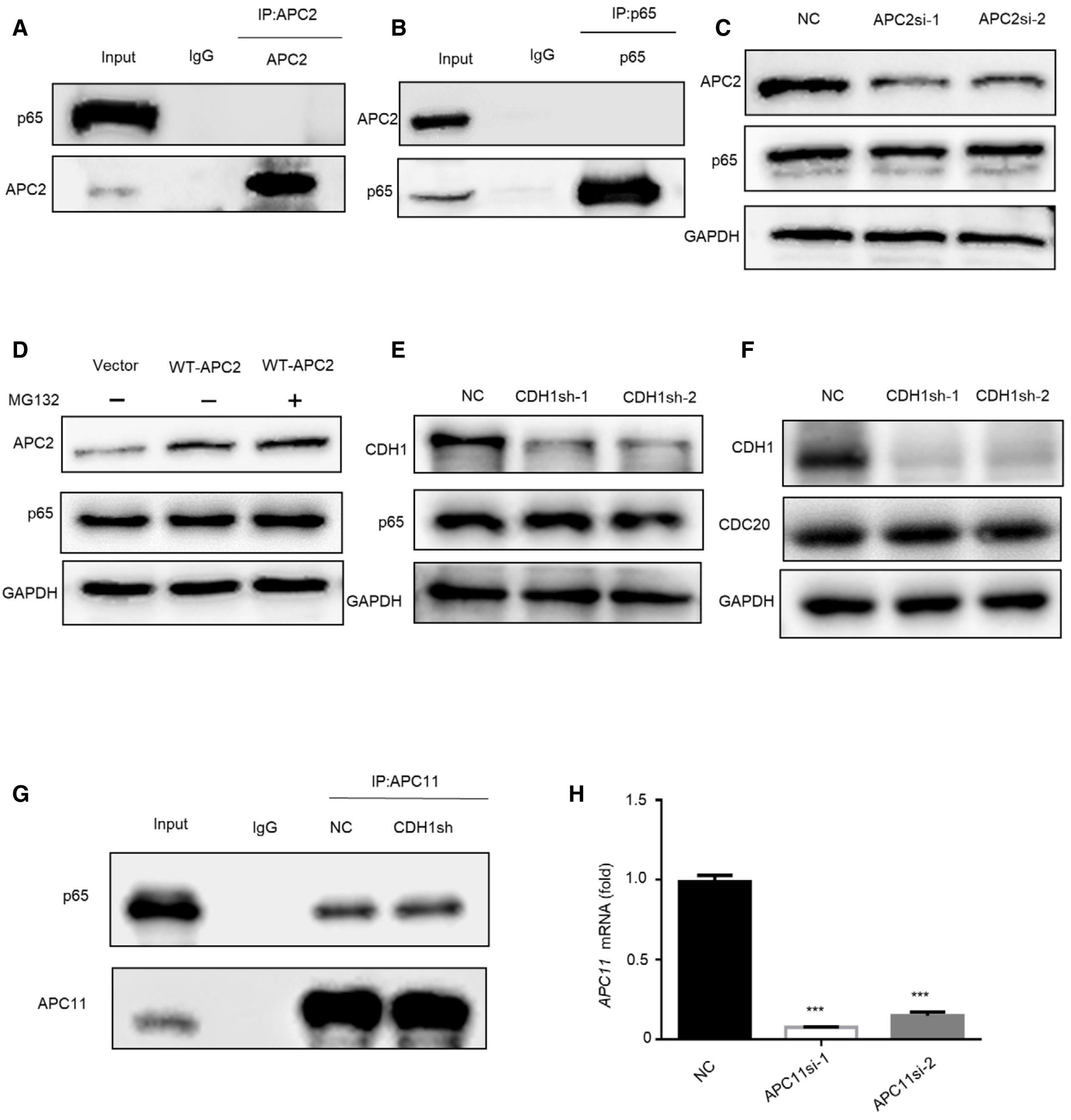

Figure EV4. CDC20 regulates p65 degradation in an APC11-dependent manner, related to Fig 6.

A, B No interaction was found in the immunoprecipitation of p65 and APC2 in HEK293T cells.
C, D The expression of p65 remained stable under the knockdown (C) or overexpression (D) of APC2 in HEK293T cells determined by Western blot analyses.
E, F No change of p65 protein (E) and CDC20 protein (F) were seen in NC and CDH1sh HEK293T cells.
G The interaction of APC11 and p65 remained stable in NC and CDH1sh HEK293T cells.
H The knockdown efficiency of APC11 in hBMSCs was determined by qRT–PCR ($n = 6$).

Data information: Data are displayed as mean ± SD and show one representative of $n \geq 3$ independent experiments with three biological replicates. Statistical significance was calculated by one-way ANOVA followed by a Tukey's post hoc test and defined as ***$P < 0.001$.

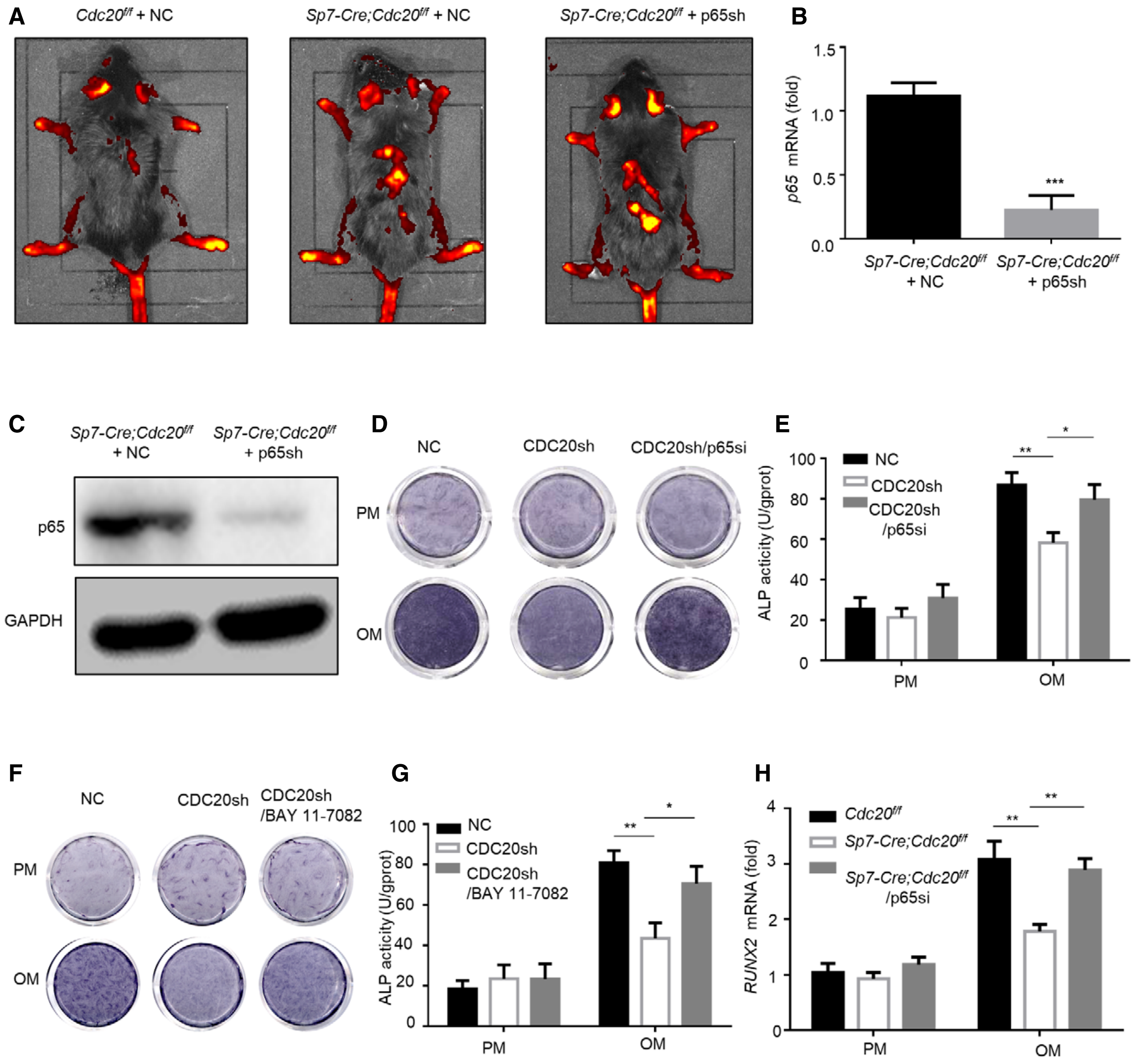

Figure EV5. CDC20 regulated osteogenic differentiation of BMSCs in a p65-dependent manner, related to Fig 8.

A Fluorescent staining of lentiviruses injected from tail intravenously.

B, C The efficiency of p65 knockdown determined by qRT–PCR (B) and Western blot (C) of BMSCs in *Sp7-Cre;Cdc20$^{f/f}$* mice (n = 6).

D, E The ALP staining (D) and ALP quantification (E) of control and CDC20 knockdown hBMSCs after 7 days osteogenic differentiation treated with negative control or p65si RNA (n = 5).

F, G The ALP staining (F) and ALP quantification (G) of NC and CDC20sh hBMSCs after 7 days osteogenic differentiation treated with BAY 11–7,082 (n = 6).

H The expression of *RUNX2* in BMSCs from *Cdc20$^{f/f}$* mice and *Sp7-Cre;Cdc20$^{f/f}$* mice after 7 days osteogenic differentiation treated with negative control or p65si, determined by qRT–PCR (n = 5).

Data information: Data are displayed as mean ± SD and show one representative of $n \geq 3$ independent experiments with three biological replicates. Statistical significance was calculated by one-way ANOVA followed by independent two-tailed Student's *t*-tests or a Tukey's post hoc test and defined as $*P < 0.05$, $**P < 0.01$, $***P < 0.001$.

 © 2021 The Authors
