# Supplementary figures and images for: CDC20 promotes bone formation via APC/C dependent ubiquitination and degradation of p65
Source: EMBO Rep. 2021 Aug 12;22(9):e52576. doi: 10.15252/embr.202152576 (PMC8419691; doi:10.15252/embr.202152576)

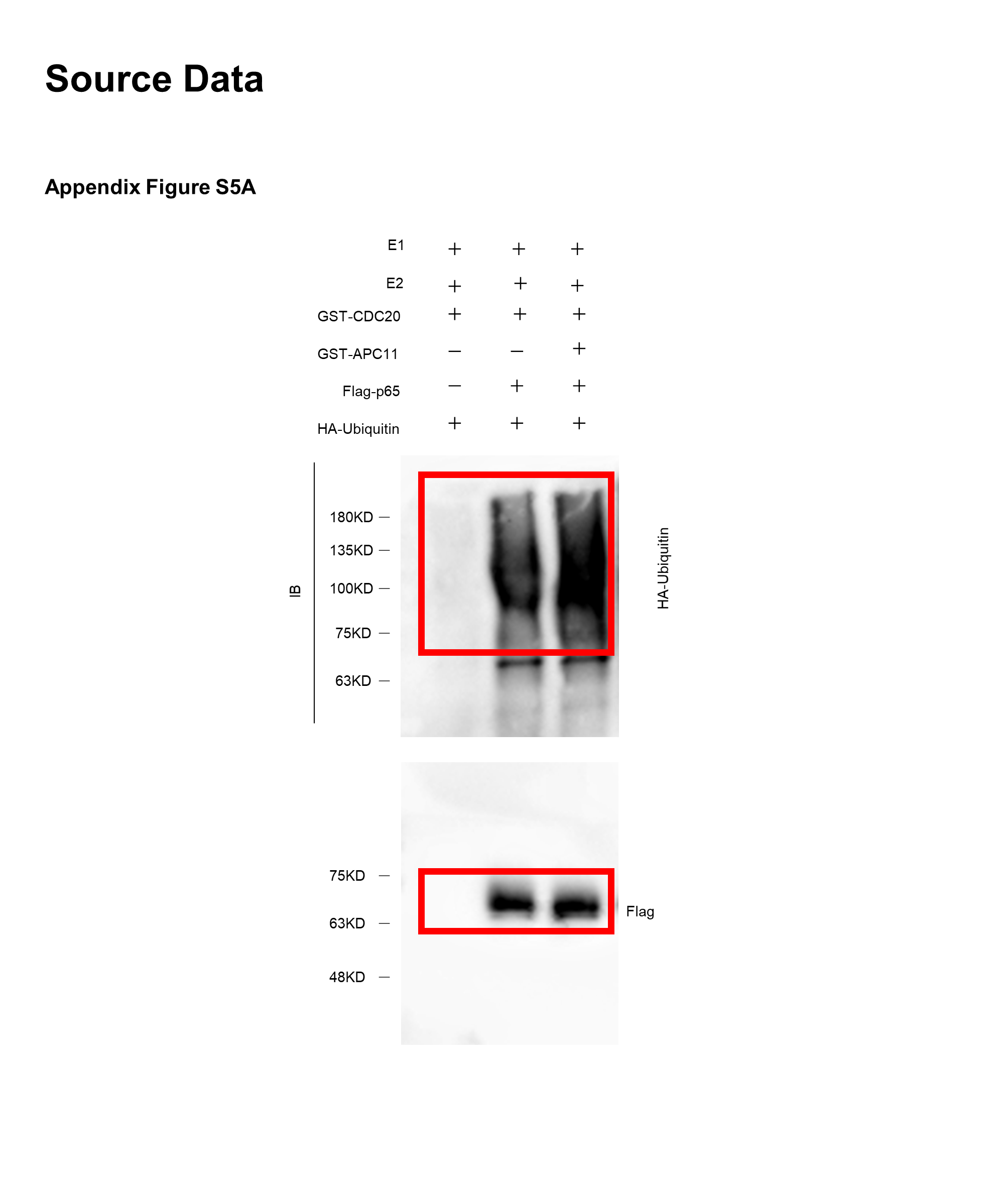

Supplement: Supplementary file 4 — Source Data for Appendix [file EMBR-22-e52576-s002.zip › embr202152576-sup-0004-SDataAppendixFigS5A.TIF]

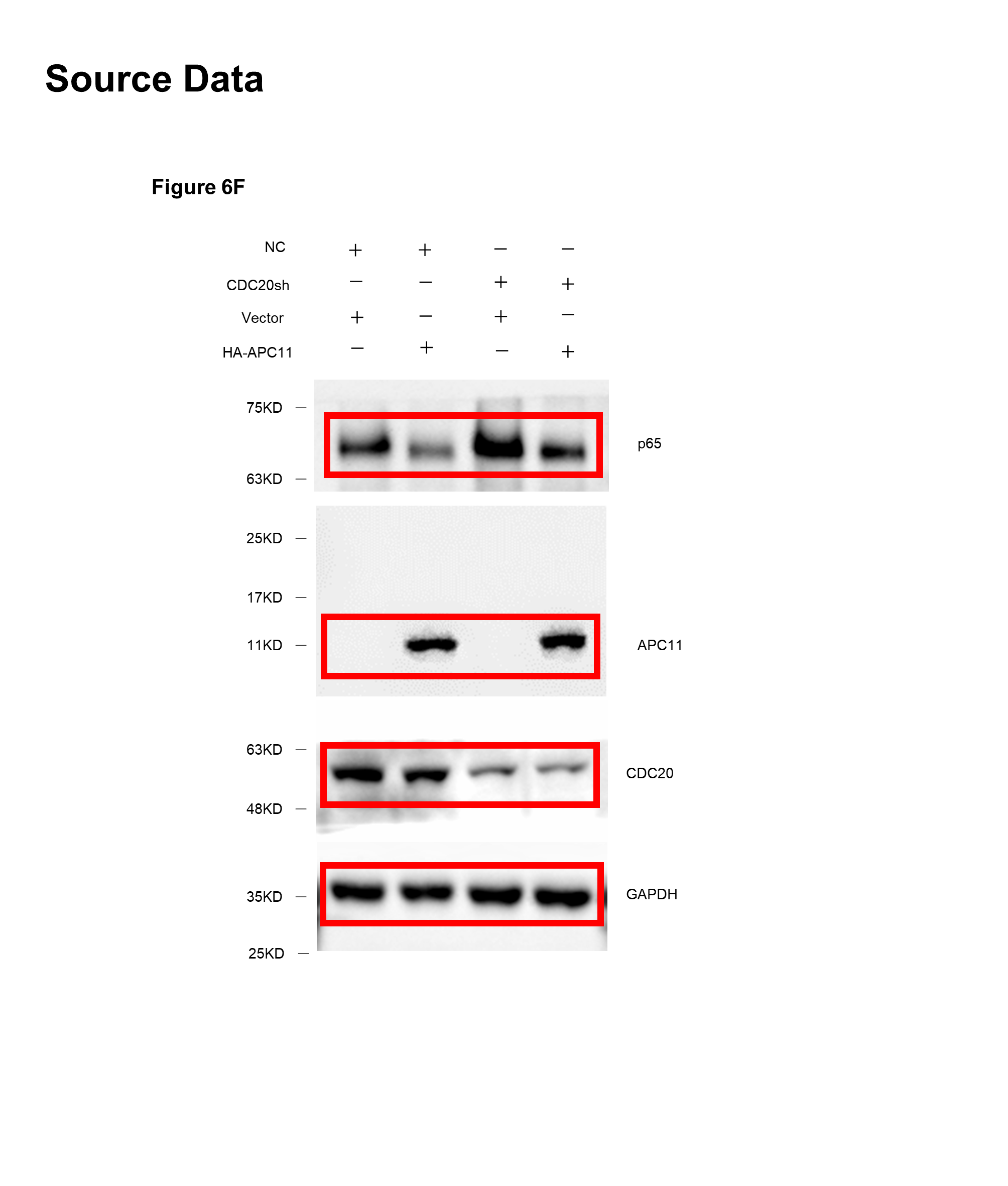

Supplement: Supplementary file 5 — Source Data for Figure 6F [file EMBR-22-e52576-s001.tif]
